# What builds the bond? Child and therapist behavior in a group intervention for aggression

**DOI:** 10.1017/S0954579425101119

**Published:** 2026-01-23

**Authors:** Robert D. Laird, John E. Lochman, Kristina L. McDonald, Caroline L. Boxmeyer, Nicole P. Powell, Lissette M. Saavedra, Lixin Qu

**Affiliations:** 1 The University of Alabama, Tuscaloosa, AL, USA; 2 Research Triangle Institute, Research Triangle Park, NC, USA

**Keywords:** Aggression, cognitive-behavioral intervention, deviant peers, group format, preadolescent

## Abstract

The study is the first to examine the effects of children’s and therapists’ in-session behaviors on later therapeutic alliance (TA; i.e., relational bond, task collaboration) as rated by children and therapists in an intervention for children with aggressive behavior. One hundred eighty children (ages 9.3–11.8; 69% male; 78% Black), screened as having aggressive behavior by teacher and parent ratings, received a 32-session group-based cognitive-behavioral intervention (Coping Power) at their schools. TA ratings were collected from children and therapists at mid- and end-of-intervention using the Therapeutic Alliance Scale for Children. Children’s and therapists’ behaviors during the first 16 sessions were coded by independent observers. Children’s negative in-session behaviors predicted lower child- and therapist-rated TA (averaged across mid- and end-of-intervention). Children’s in-session positive behaviors, at both the individual and group-wide level, predicted higher later TA. Therapists’ efforts to manage deviant behavior predicted stronger child-reported ratings of the relational bond and of child- and therapist-rated task collaboration. Exploratory analyses indicate that the effect of children’s in-session behaviors on later TA is moderated by therapists’ skills in managing the group and in managing deviant talk and behavior in sessions. Clinical and research implications of the findings are discussed.

Cognitive-behavioral interventions for children with aggressive and disruptive behavior problems, and their parents, have become a notable part of the growing catalog of evidence-based treatments, based on results from rigorous randomized clinical trials (Kaminski et al., 2024; Matthys & Lochman, 2017). One such evidence-based cognitive-behavioral intervention, the Coping Power program, has produced reductions in aggressive and disruptive behaviors for pre-adolescent at-risk children at one-year (Lochman & Wells, 2003, 2004) and three-year (Lochman et al., 2013, 2014) follow-up periods, has been adapted to include mindfulness training (Boxmeyer et al., 2023) and internet delivery (Lochman, Boxmeyer, et al., 2017), and to be delivered as an universal preventive intervention within classrooms (Muratori et al., 2017) and with adolescents (Bradshaw et al., 2025). However, the effect sizes of the behavioral outcome effects for Coping Power and related interventions are typically only in the small-to-moderate range for clinical and school-based samples (McCart & Sheidow, 2016; Sukhodolsky et al., 2004).

There has been an increasing interest in rigorously understanding process effects in interventions with adults, with recognition that aspects of the therapeutic process, such as therapeutic alliance, can vary across clients and have a “necessary but not sufficient” effect on cognitive-behavioral intervention outcomes (Kazantzis, 2018). However, this remains an underdeveloped area in child intervention research, as evidence-based interventions have largely focused on the therapists’ adherence to the content of the intervention manual and relatively ignored interpersonal processes (Crawford et al., 2018). The modest success of evidence-based interventions with difficult-to-treat children and adolescents can thus be due in part to a narrow focus only on the integrity of delivery of manual-based intervention tasks, rather than on interpersonal processes as well (Cunningham et al., 2023). This lack of focus on process issues in interventions with children is likely due in part to an underlying assumption that therapists possess basic facilitative skills that could assist all children. However, research on process factors in intervention with anxious youth has found that therapist actions during sessions, including factors such as coaching style and critical behavior, can affect client motivation, involvement, engagement and the therapeutic alliance (Crawford et al., 2018).

## Therapeutic alliance

The therapeutic alliance (TA) refers to the engagement between therapist and client in the work of therapy (Horvath & Symonds, 1991). TA has two primary elements: (a) relational bond, a positive affective relationship between therapist and client, and (b) task collaboration, or the collaborative inherent agreement between therapists and clients on the tasks and goals of intervention (Shirk & Karver, 2003). Although research examining TA in children often uses a single score collapsing these two elements, the two TA elements differentially relate to intervention-targeted mechanisms (Albaum et al., 2023). A positive TA may motivate the client to be actively engaged in therapy activities and to remain in treatment (de Haan et al., 2013; Kendall et al., 2009).

There has been a growing focus on the relation between TA and child outcomes, indicating that TA has unique effects on outcomes above and beyond the intervention effect itself. A meta-analysis of 99 studies found a small but significant effect size (*r* = .17) for the association between child-therapist TA and subsequent child outcomes following treatment (Roest et al., 2023b). Even though the overall associations are modest, the alliance-outcome association was strongest for children with externalizing problems, indicating that TA is clinically important and can be considered an important factor in child and adolescent psychotherapy (Roest et al., 2023a). TA has been primarily investigated in individual therapy sessions for children with externalizing problems. For example, Kazdin and Durbin (2012) found that higher quality TA predicted greater therapeutic change at the end of individual cognitive-behavioral treatment for children with oppositional and antisocial behavior. Similarly, Mattos et al. (2017) found that TA predicted reductions in adolescents’ delinquent behavior for youth seen in individual and family therapy. Examining observed ratings of TA during individual administration of the Coping Power program’s child component (the individual version of the group-based Coping Power program examined in the current study), Mitchell and colleagues (2021) found that higher levels of relational bond in early sessions predicted greater reductions in teacher-rated externalizing behavior.

Most research on TA with children has been in the context of individual therapy with limited investigation into whether TA has equally important effects on outcomes when children are seen in group therapy. It is important to consider how TA develops in group interventions because school-based group treatment is a common way for children to receive mental health services, especially for children who may not have the family resources to access private individual therapy. Group processes could conceivably either enhance or diminish the TA. Establishing TA in group intervention can be difficult because the therapist must develop alliances with multiple children at the same time. For children with anxiety disorders, TA has been found to be more related to elimination of anxiety diagnoses for children seen in group therapy than in individual therapy (Bjaastad et al., 2023).

Research on development of TA in group interventions for children with externalizing behavior problems is very limited but even more critical because of concerns about potential impulsivity, disruptiveness, contagion effects, and deviant peer processes among children with these problems. As a result, group intervention with these children can be frustrating for therapists and likely requires therapists to use more deliberate behavioral management methods which may make collaborative development of TA difficult. In a study of children identified for intervention by elevated levels of teacher- and parent-rated aggression, higher levels of therapist-rated relational bond and task collaboration, assessed at mid-intervention and end-of-intervention, predicted reductions in externalizing behavior at a one-year follow-up after the Coping Power intervention (Lochman et al., 2024). Importantly, TA was found to be equally predictive of intervention outcomes for children seen individually and for children seen in groups (Lochman et al., 2024), indicating the functional role of TA in group therapy for these children.

## Therapist and child in-session behavior as predictors of therapeutic alliance

Given the importance of TA in predicting children’s outcomes following intervention, reviews of the TA literature have argued for increased attention on how therapist and child behaviors which occur during sessions could interact and contribute to the development of TA (Ryan et al., 2023). TA is a dynamic interpersonal process and likely is affected by both therapist and child behaviors. Although the existing literature largely addresses therapist or child behavior separately, as described in the following sections, the current study will consider the behaviors of both persons in the alliance in combination. Only a few studies have considered these in-session behaviors in group intervention for children, and none have examined children’s behavior in the context of their peers’ behavior in the groups. Plausibly a given child’s behavior could be perceived differently and differentially affect TA, depending on whether the child’s behavior was similar to or in contrast to the behavior of others in the group.

### Therapist in-session behaviors

In treatment with adults, stronger therapeutic alliances have been evident with therapists who displayed more empathy and focus on feelings (HaRim Ahn & Kivlighan, 2021) but have been unrelated to therapists’ directiveness (Watson & McMullen, 2005). In family treatment with children and adolescents, weaker alliances with family members have occurred when therapists competitively respond to family members and struggle for interpersonal control (De la Pena et al., 2012) and when therapists provide low levels of engagement and emotional connection behaviors (Welmers-van de Poll et al., 2021).

Therapist behaviors are also related in important ways to TA in individual cognitive-behavioral therapy (CBT) with children (Ryan et al., 2023). In CBT with adolescents with depressive disorders, therapists who had higher levels of attending to adolescents’ experience and motivation and lower levels of structure in initial sessions were found to have greater levels of client involvement in subsequent sessions (Jungbluth & Shirk, 2009). For children receiving trauma-focused CBT, therapists who used more rapport-building behaviors in early sessions, including eliciting children’s accounts of their experiences, had higher levels of youth-reported TA in later sessions (Ovenstad et al., 2020). Similarly, the TA in CBT for children with anxiety disorders has been found to be stronger when the therapists use a more collaborative coaching style in the session, use less rigid structuring, and are less critical (Crawford et al., 2018; Fjermestad et al., 2021).

Despite the increasing understanding of how therapist behavior affects TA for children with internalizing problems, there is a lack of research on how therapist behaviors affect children with externalizing behavior problems or how the therapist behavior influences the development of TA in group therapy. The characteristic defiance, overactive behaviors and poor emotional regulation of children with externalizing behavior problems may affect the development of a functional TA through different therapist in-session behaviors, especially in the context of group intervention. In a study that examined how therapist behaviors related to behavioral outcomes following the Coping Power cognitive-behavioral intervention, group therapists’ clinical skills (displaying warmth and flexibility, not reacting in irritable ways), but not group and behavioral management skills predicted greater reductions in children’s conduct problems at a one-year follow-up (Lochman, Dishion, et al., 2017) and externalizing behaviors at a four-year follow-up (Lochman et al., 2019). Although these clinical skills affect children’s outcomes, it is not clear if they affect critical intervention processes such as TA. It is plausible that the therapists’ clinical skills in early intervention sessions may also predict the development of therapists’ and children’s perceptions of stronger relational bonds and task collaboration but that has not been examined.

### Child in-session behaviors

The way children behave in session is likely to affect the development of TA, yet very little research has examined this issue. Some studies have examined how children’s pre-treatment characteristics such as emotion regulation (Albaum et al., 2020), depression (Labouliere et al., 2017), and social and intellectual competence (Kazdin & Durbin, 2012) have predicted TA during subsequent treatment. In a rare study specifically examining children’s in-session behaviors, children who initiated very little verbalization in early sessions of trauma-focused CBT had lower TA at mid-treatment, as rated by observers. However, if the therapists used an active trauma-eliciting approach, then the TA was enhanced even for children with low initial verbalization rates (Ovenstad et al., 2020).

No studies have examined how in-session behaviors of children treated for externalizing behavior problems might affect the development of their TA. In a study noted previously (Lochman, Dishion, et al., 2017), children’s positive and negative in-session behaviors were examined in conjunction with therapist behaviors as predictors of children’s behavioral outcomes through follow-up periods, after their involvement in the Coping Power group-based intervention. Higher rates of children’s negative in-session behaviors (e.g. deviant talk, off-task and inattentive behavior, verbal or physical aggression) predicted higher rates of aggressive and conduct problems behaviors at a one-year follow-up (Lochman, Dishion, et al., 2017). Although these child in-session behaviors affect children’s outcomes, it is not clear if they affect the development of the TA.

## The present study

This study addresses an emerging area of interest in research on process factors in child interventions and focuses on the unexplored ways in which therapist and child in-session behaviors could affect the development of the TA in a group-based cognitive-behavioral intervention (the Coping Power child component) for children at risk because of high levels of aggressive behavior. There have been several notable studies of how in-session therapist behaviors predict the development of the TA in individual interventions with children with internalizing problems. However, there is no literature on TA that examines, with child clients who have difficult-to-treat externalizing behavior problems, how child and therapist in-session behaviors in group therapy affect TA. To address this issue, this study was conducted as a secondary analysis of the portion of the sample from a study by Lochman and colleagues (Lochman et al., 2015) who received the traditional group format of the Coping Power program. The present study used data from prior studies about therapist and child in-session behaviors (Lochman, Dishion, et al., 2017), from sessions during the first half of the program, to predict trait-level TA (Zilcha-Mano & David-Sela, 2022) assessed at mid- and end-of-intervention (Lochman et al., 2024). Trait-like levels of TA are calculated by averaging across TA ratings at different points in treatment (in our case at mid-intervention and end-of-intervention, the only two time points when TA was assessed) and indicate stable patient differences in alliance strength (Zilcha-Mano & David-Sela, 2022). In contrast state-like TA indicates within-patient changes in TA during treatment. The conceptualization of TA as being trait-like or state-like has become an important area of research in treatment with adults (Zilcha-Mano & David-Sela, 2022) but had rarely been examined in intervention with children. Lochman and colleagues (2024) found that it was the trait-like TA, and not state-like TA (which indicated variation in TA between mid- and end-of-intervention), that predicted children’s later behavioral outcomes, and thus the present study focuses on trait-like TA.

Although the present study is exploring a novel topic, several hypotheses were advanced based on related research. First, it was hypothesized that higher rates of positive and engaged in-session child behavior (based on the study by Ovenstad et al., 2020) and lower rates of negative child behavior (based on the study by Lochman, Dishion, et al., 2017) would predict higher rates of the two domains of therapist- and child-reported relational bond and task collaboration. Second, it was hypothesized that high rates of therapists engaged clinical skills (based on the study by Welmers-van de Poll et al., 2021, and by the studies by Lochman, Dishion, et al., 2017; Lochman et al., 2019) would predict higher rates of the two domains of TA.

Because of their exploratory nature, there are also two research questions examined without specific hypotheses. The first research question examines a neglected issue of whether group-wide levels of children’s positive and negative in-session behaviors predict trait TA. Group therapy is a dramatically different intervention format than individual therapy, and the development of TA could be influenced by the aggregate behavior of individual children in the group. Perceptions of children’s behavior is affected by the behavior of children in their environment (e.g. Barth et al., 2004), but because group-level behavior has not been examined in prior TA research it is not clear if group-level behavior influences the development of TA. The second research question is the most important part of this study and asks whether child and therapist behavior in early sessions interact to form the TA. Ryan and colleagues (2023) recommended that research should move beyond looking at child and therapist behavior as singular predictors of TA and instead explore how the mutual and dynamic interactions between child and therapist behaviors affect the evolution of the TA. For example, the impact of children’s positive or negative behavior on the development of TA may depend on whether the therapist has sufficient clinical skills to leverage positive behavior into an effective alliance or whether the therapist is adept at managing negative behavior so that it does not interfere was the development of the alliance.

## Methods

### Participants

Participants were part of a larger randomized controlled trial comparing group versus individual formats of the Coping Power program (Lochman et al., 2015, 2017). The full sample included 360 fourth-grade students at twenty participating schools. Students who scored in the top 25^th^ percentile on a teacher screener of aggressive behavior (Dodge et al., 1997) and in the average range or above on parent-rated aggression on the Behavior Assessment System for Children (BASC: Reynolds & Kamphaus, 1992) were recruited to participate. Students were randomly assigned to group or individual Coping Power by school. The intervention was implemented during spring of 4^th^ grade through the end of 5^th^ grade.

The current study focused only on children who participated in group-based Coping Power, which included 180 students. The mean age of these children at the time of recruitment was 10.2 years (range 9.3 to 11.8). About two-thirds of the participants were boys (68.9%) and about one-third girls (31.1%). The participants identified as Black/African American (77.8%), White/European American (17.8%), Hispanic (1.7%), and “Other” (2.8%). The distribution of reported family income was as follows: 3.9% reported no income, 27.8% less than $15,000, 32.2% between $15,000 and $29,999, 16.7% between $30,000 and $49,999 and 19.4% greater than $50,000. As expected, given the screening procedure targeting children with moderate to high aggression, the sample had elevated externalizing behavior problems at baseline. Teacher and parent ratings on the BASC Externalizing Composite fell in the at-risk range (T scores between 60 and 69) for 33.9% and 19.4% of the sample, respectively, with an additional 32.2% and 22.8% of the sample having BASC Externalizing Composite scores that exceeded the clinical cutoff (T score greater than or equal to 70).

The Coping Power intervention was delivered by 20 intervention staff. Each group was led by a primary therapist (2 doctoral level, 17 psychology doctoral students, and 1 bachelor-level staff) as well as a co-therapist (which was typically a school counselor, graduate student, or research assistant). The primary therapists included 17 female (85%) and 3 male (15%) therapists. Fifteen identified as White/European American (75%), 4 as Black/African American (20%), and 1 as White-Hispanic (5%).

### Procedure

Coping Power is a manualized skills training program that targets key social-cognitive deficits in children with aggressive behavior. While the full Coping Power program includes both child (Lochman et al., 2008) and parent (Wells et al., 2008) components, only the child component was implemented in the current study. The intervention included 32 weekly group meetings, which lasted approximately 50–60 minutes each and were held during the school day. The primary Coping Power therapists participated in an intensive 2-day training that covered the background and research on the Coping Power program, a session-by-session overview of the curriculum, and guidance on engaging children in the intervention and behavior management strategies. While the intervention was being implemented, weekly supervision meetings were held during which therapists provided updates on students’ progress, material to be delivered in upcoming sessions was reviewed, and issues that arose during Coping Power group meetings were discussed and planned for. Therapists also received monthly supervisory feedback on video-recorded sessions to ensure high fidelity and quality of program implementation.

All study procedures were approved by the University of Alabama Institutional Review Board. For comprehensive information about the study, participants, and intervention procedures, see Lochman et al. (2015) and Lochman, Dishion, et al. (2017).

### Measures

#### Therapeutic alliance

The Therapeutic Alliance Scale for Children (TASC; Shirk & Saiz, 1992) was administered to assess child and therapist perspectives on the TA at the midpoint (halfway through the intervention, after the first 16 sessions) and endpoint (at the last session) of the Coping Power intervention. Children and therapists each completed parallel versions of the measure. The TASC has 12 items and uses a Likert scale ranging from 1 = Not true to 4 = Very much true.

The *relational bond* subscale captured the affective bond and experiences between the child and therapist and was assessed with six items such as, “The child looks forward to Coping Power meetings”/“I look forward to meeting with my Coping Power leaders” (therapist-report α_midpoint_ = 0.92, α_endpoint_ = 0.92; child-report α_midpoint_ = 0.83, α_endpoint_ = 0.79). The *task collaboration* subscale assessed how the child worked together with the therapist on therapeutic tasks and was assessed with six items such as, “The child is able to work well with you on dealing with his/her problems/issues”/ “I think that my Coping Power leaders and I work well together on dealing with my problems” (therapist-report α_midpoint_ = 0.90, α_endpoint_ = 0.91; child-report α_midpoint_ = 0.57, α_endpoint_ = 0.66).

As described by Lochman and colleagues (2024), trait-like scores reflecting child and therapist perspectives on the relational bond and task collaboration were calculated as the mean of the midpoint and endpoint scores (on the respective child and therapist reports). Correlations between midpoint and endpoint ratings were as follows: child report of relational bond = 0.52, child report of task collaboration = 0.48, therapist report of relational bond = 0.80, and therapist report of task collaboration = 0.65 (all *p* < .001).

#### Cognitive–Behavioral group coding system

As described in Lochman, Dishion, and colleagues (2017), a behavioral coding system was developed to rate child and therapist behavior during Coping Power group sessions. The coding system utilized a macro rating scale in which the interactions between each participant and all other participants were recorded in a matrix (Boxmeyer et al., 2015). We used child and therapist behavior coded in the first 16 sessions of the intervention to ensure that the behaviors were observed prior to the assessment of TA. Separate ratings were made for the behavior of each child participant and each group therapist during the first ten minutes, middle ten minutes, and last ten minutes of each session, and the ratings (16 sessions with three ratings per session) were aggregated for analyses. Items were rated on a 5-point Likert scale ranging from 0 = not at all to 4 = very often.

Child-rated behaviors included positive child behaviors (e.g., showing involvement and interest in group discussion and activities; initiating positive and friendly interactions with other group members; other children initiating reciprocal positive and friendly interactions toward this child) and negative child behaviors (e.g., deviant talk, exhibiting off-task, inattentive behavior; engaging in silly or disruptive behavior; demonstrating a negative, hostile attitude; exhibiting verbal or physical aggression; and appearing to trigger these negative behaviors in other group members). Children’s deviant talk and behavior was initially coded separately but was found to strongly correlate with other negative child behaviors (*r* = .81, *p* < .001), thus these items were included in the overall negative behavior code. In the current analyses, *positive and negative child behaviors* were calculated at both the individual and group levels.

Therapist behaviors fell into four categories. These included: *Behavior Management Strategies* (e.g., therapist sets clear expectations and rules for group behavior; enforces rules for group behavior effectively; provides strategic reinforcement for desired behaviors, provides consequences for rule violations, adheres to an agenda/manages group time effectively; quiets the children and elicits their attention effectively), therapist’s use of *Teaching Strategies* (e.g., provides clear rationale for new topics and activities; provides clear instructions; reviews key teaching points; actively assesses children’s comprehension, creates “teaching moments,” therapist elaborates the content beyond the manualized material), and *Clinical Skills* (e.g., therapist’s tone is warm and positive; therapist demonstrates professionalism in dress, behavior, and level of self-disclosure; therapist is overly rigid-reverse scored; therapist appears frustrated, angry or irritable-reverse scored). The scales for *Behavior Management Strategies* and *Teaching Strategies* were highly correlated in this sample (*r* = .85, *p* < .001) and were thus aggregated into one code for the analyses, which was labelled *Group Management Skills.* Our three base subscales (Clinical Skills, Behavior Management Strategies, Teaching Strategies) are conceptually similar in certain central ways to other rating systems that have examined adult behavior with groups of children in school settings, such as the Classroom Assessment Scoring System (CLASS; Pianta et al., 2008). Research with the CLASS has found that related dimensions of teachers’ behavior, including emotional support, classroom organization, and instructional support, are related to children’s academic outcomes.

*Managing Deviant Talk and Behavior.* Therapists were coded as more effective at managing deviant talk and behavior if they made an overt effort to discourage the talk or behavior, re-direct the conversation, or re-establish appropriate norms (e.g., therapist tries to discourage deviant behavior or counter norm talk by redirecting or changing the subject; therapist re-establishes appropriate norms following occurrences of deviant behavior or counter norm talk). Therapists were coded as less effective at managing deviant talk and behavior if they showed interest or paid attention without intervening or inadvertently encouraged the deviant talk or behavior (e.g., therapist encourages counter norm talk by showing interest, asking questions, or simply listening and paying attention – reverse scored; therapist inadvertently encourages prolonged deviant talk or behavior while attempting to illustrate a teaching topic – reverse scored). Thus, the Managing Deviant Talk and Behavior included, on the one hand, therapists’ active efforts to redirect group back to task, and, on the other hand, therapists’ avoidance of encouraging deviant talk themselves through their own positive attention and comments about the deviant statements. The three therapist behavior scales used in this study are *Managing Deviant Talk and Behavior*, *Clinical Skills*, and *Group Management Skills*.

### Analysis plan

Multi-level models were used to test the primary hypothesis that TA is linked to child behavior, therapist behavior, and interactions involving child and therapist behavior (see supplementary materials for the full multi-level model specification). Child behavior was modeled as an individual level variable with group behavior and therapist behavior modeled as group level variables. Only the intercept was modeled as a random effect because exploratory analyses specifying random slopes typically failed to converge and/or showed minimal and non-significant variability in individual positive and negative behavior slopes.

The analysis included seven child/therapist behaviors and eight interaction terms. Specifically, TA was predicted by individual positive behavior, individual negative behavior, group positive behavior, group negative behavior, therapist group management skills, therapist clinical skills, and therapist skill at managing deviant talk and behavior and the following interaction terms: individual positive behavior × group positive behavior, individual negative behavior × group negative behavior, individual positive behavior × therapist group management skills, individual positive behavior × therapist clinical skills, individual positive behavior × therapist managing deviant talk and behavior, individual negative behavior × therapist group management skills, individual negative behavior × therapist clinical skills, individual negative behavior × therapist managing deviant talk and behavior. We did not adjust p-values for multiple tests to maximize statistical power because more statistical power is required to find interaction effects than main effects (McClelland & Judd, 1993). Statistically significant interactions were interpreted by calculating and plotting simple slopes of the individual level variable at high (+1SD), mean (0 SD), and low (-1 SD) levels of the group-level variable according to the procedures described by Preacher et al. (2006).

## Results

### Exploratory analyses

Five sets of exploratory analyses were conducted to ensure that the primary analyses were robust to maximize the potential for replication. TA was assessed at intervention mid-point and endpoint whereas children’s and therapists’ behaviors were assessed multiple times during each session. Our first set of exploratory analyses (presented in Supplementary Table 1) calculated intra-class correlations for child and therapist behaviors to determine if there was variability to predict within sessions, across sessions, across children, and/or across groups. Results indicated that variability in child and therapist behavior was primarily across children (17.9%–19.2%) and across groups (2.4%–32.3%) rather than across sessions (0.1%–5.6%) or portion of the session (1%–1.1%), so we focused on predicting TA from individual and group level measures of child and therapist behavior that aggregated within and across sessions. We aggregated across the first 16 sessions to ensure that child and therapist behavior variables measured behaviors that occurred before TA was assessed.

The second set of analyses sought to evaluate bivariate associations among and between child behaviors, therapists’ behaviors, and the indices of TA (See Supplementary Table 2). The third set of analyses tested individual child and therapist behaviors as predictors of TA (see Supplementary Table 3) and compared those results with results of analyses combining multiple child and therapist behaviors in the same model. Finding from the univariate analyses largely replicated in the multivariate models (presented below), and the multivariate models also allowed us to test the individual × group and child × therapist interactions of interest, so we emphasize the multivariate analyses. The fourth set of analyses tested child and therapist behaviors in the first 16 sessions as predictors of TA at intervention mid-point, intervention endpoint, and TA change from mid-point to endpoint (i.e., intervention endpoint controlling for intervention mid-point) (see Supplementary Table 4). Results predicting mid-point and endpoint TA were similar, with minimal evidence that behavior in the first 16 sessions predicted change from mid-point to endpoint (although the models for child-reported TA failed to converge), so we combined the two TA measures to produce a measure of trait-level TA and emphasize the prediction of trait-level TA from child and therapist behavior in the first half of the intervention in the primary analyses. Finally, the fifth set of analyses included the main effects from the primary multi-level model, but not the interaction effects (see Supplementary Table 5). Because many of the main effects were qualified by significant interactions, we present results from the multivariate multi-level models including interaction terms in the next section.

### Multi-level models

The multivariate multi-level models provide evidence of the extent to which reports of TA could be predicted by child behaviors, therapists’ behaviors, and the interactions between individual and group behavior and between child and therapists’ behaviors. Table 1 presents parameter estimates when predicting child-reported TA and Table 2 presents parameter estimates when predicting therapist-reported TA.

**Table 1.** Child-reported therapeutic alliance predicted by child and therapist behavior

	Relational bond					Task collaboration				
Predictor	β	*s.e.*	*df*	*p*	95% CI	β	*s.e.*	*df*	*p*	95% CI
Individual positive (IP)	0.225	0.241	134.952	.351	[−0.251, 0.701]	0.271	0.228	136.080	.235	[−0.179, 0.721]
Individual negative (IN)	−1.141	0.506	136.624	.026	[−2.143, −0.140]	−0.904	0.477	136.770	.060	[−1.847, 0.040]
Group positive (GP)	0.320	0.376	45.562	.398	[−0.436, 1.077]	0.024	0.326	60.560	.942	[−0.628, 0.676]
Group negative (GN)	0.714	0.722	39.573	.329	[−0.746, 2.174]	0.113	0.622	55.139	.856	[−1.133, 1.359]
Group management skills (GM)	−0.296	0.182	20.801	.118	[−0.674, 0.082]	−0.115	0.148	25.578	.443	[−0.421, 0.190]
Clinical skills (CS)	0.015	0.493	18.485	.975	[−1.018, 1.049]	0.199	0.398	23.106	.622	[−0.625, 1.024]
Managing deviant talk & behavior (MD)	3.027	1.463	19.543	.052	[−0.028, 6.083]	3.045	1.187	23.365	.017	[0.593, 5.498]
IP × GP	0.021	0.133	69.016	.878	[−0.245, 2.86]	−0.050	0.117	60.443	.669	[−0.284, 0.184]
IN × GN	−1.134	0.496	82.945	.025	[−2.120, -0.147]	−0.431	0.443	82.588	.333	[−1.311, 0.450]
IP × GM	0.037	0.071	116.141	.603	[−0.103, 0.177]	0.048	0.064	106.537	.458	[−0.080, 0.175]
IP × CS	−0.166	0.236	62.589	.485	[−0.637, 0.306]	−0.176	0.206	57.783	.397	[−0.589, 0.237]
IP × MD	−0.651	0.567	41.719	.257	[−1.796, 0.493]	−0.626	0.484	40.484	.203	[−1.603, 0.351]
IN × GM	−0.244	0.156	91.676	.122	[−0.554, 0.067]	−0.210	0.140	83.484	.138	[−0.488, 0.069]
IN × CS	−0.638	0.462	130.509	.170	[−1.552, 0.276]	−0.258	0.426	120.944	.546	[−1.101, 0.585]
IN × MD	4.767	1.647	127.996	.004	[1.508, 8.027]	2.288	1.514	116.818	.134	[−0.711, 5.287]

**Table 2.** Therapist-reported therapeutic alliance predicted by child and therapist behavior

	Relational bond					Task collaboration				
Predictor	β	*s.e.*	*df*	*p*	95% CI	β	*s.e.*	*df*	*p*	95% CI
Individual positive (IP)	1.038	0.206	131.256	<.001	[0.631, 1.444]	0.988	0.195	132.707	<.001	[0.602, 1.374]
Individual negative (IN)	−1.886	0.430	131.703	<.001	[−2.737, −1.035]	−1.961	0.408	132.510	<.001	[−2.767, −1.155]
Group positive (GP)	−0.404	0.315	33.460	.208	[−1.045, 0.236]	−0.330	0.295	42.599	.270	[−0.926, 0.266]
Group negative (GN)	0.301	0.562	25.296	.598	[−0.857, 1.458]	0.449	0.526	33.087	.400	[−0.622, 1.519]
Group management skills (GM)	−0.562	0.149	13.165	.002	[−0.883, −0.240]	−0.559	0.139	17.232	<.001	[−0.851, −0.267]
Clinical skills (CS)	0.062	0.393	10.528	.878	[−0.808, 0.931]	−0.031	0.364	13.844	.933	[−0.814, 0.751]
Managing deviant talk & behavior (MD)	2.187	1.216	13.124	.095	[−0.438, 4.812]	3.253	1.131	17.173	.010	[0.869, 5.637]
IP × GP	−0.237	0.110	45.837	.037	[−0.458, −0.015]	−0.249	0.103	53.220	.020	[−0.456, −0.042]
IN × GN	−0.162	0.412	69.964	.695	[−0.983, 0.660]	−0.431	0.388	78.764	.270	[−1.204, 0.341]
IP × GM	0.179	0.060	101.959	.004	[0.060, 0.298]	0.134	0.057	106.912	.020	[0.021, 0.247]
IP × CS	0.029	0.192	37.698	.879	[−0.359, 0.418]	−0.058	0.180	44.694	.750	[−0.420, 0.305]
IP × MD	−0.023	0.470	25.295	.961	[−0.990, 0.944]	0.138	0.440	31.198	.755	[−0.758, 1.035]
IN × GM	−0.122	0.133	68.100	.362	[−0.387, 0.143]	−0.209	0.125	76.115	.099	[−0.458, 0.040]
IN × CS	0.389	0.389	119.755	.319	[−0.381, 1.159]	−0.134	0.368	121.549	.717	[−0.862, 0.594]
IN × MD	3.184	1.430	111.622	.028	[0.351, 6.017]	1.563	1.352	114.561	.250	[−1.114, 4.240]

#### Child-reported TA

**Figure 1.**
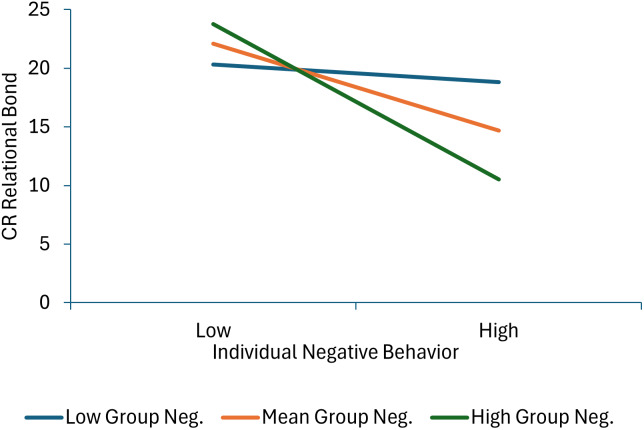
This figure decomposes the interaction between individual and group negative behavior when predicting child-reported relational bond.

**Figure 2.**
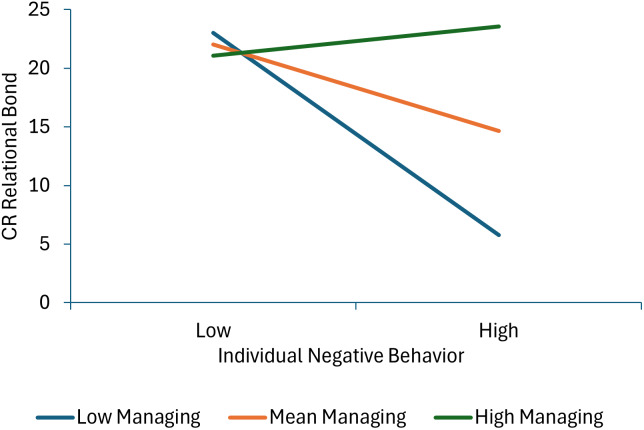
This figure decomposes the interaction between individual negative behavior and managing deviant behavior when predicting child-reported relational bond.

Three terms were statistically significant in the model predicting child-reported relational bond: individual negative behavior, the individual negative behavior × group negative behavior interaction, and individual negative behavior × managing deviant talk and behavior interaction. None of the child or therapist behaviors during the first half of the intervention uniquely predicted child-reported task collaboration scores. As shown in Figure 1 (parameter estimates for all significant interactions are presented in Supplementary Table 6), more individual negative behavior was associated with lower levels of relational bond at mean levels of group negative behavior and at high levels of group negative behavior, but not at low levels of group negative behavior, suggesting that more individual negative behavior did not impact TA if the group engaged in little negative behavior, but increasingly and negatively impacted TA as the group engaged in more negative behavior. As shown in Figure 2, individual negative behavior was associated with lower levels of relational bond when therapists engaged in average or low levels of managing deviant talk and behavior, but not when the therapist engaged in high levels of managing deviant talk and behavior.

#### Therapist-reported TA

**Figure 3.**
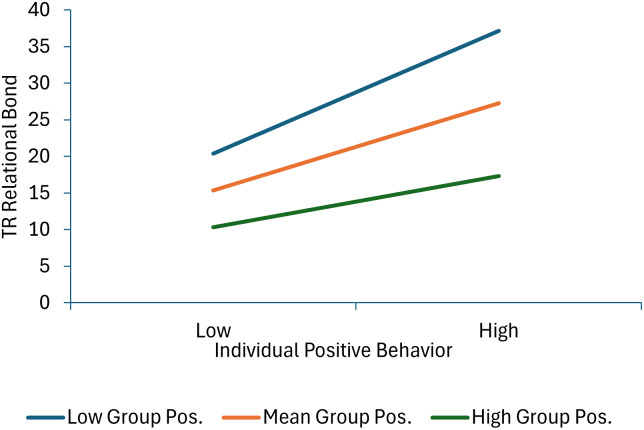
This figure decomposes the interaction between individual and group positive behavior when predicting therapist-reported relational bond.

**Figure 4.**
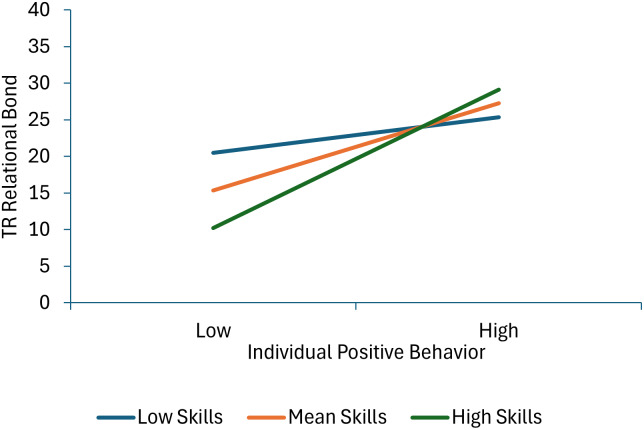
This figure decomposes the interaction between individual positive behavior and group management skills when predicting therapist-reported relational bond.

**Figure 5.**
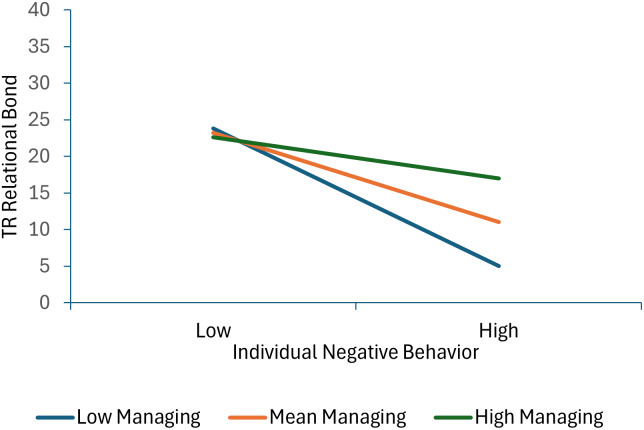
This figure decomposes the interaction between individual negative behavior and managing deviant behavior when predicting therapist-reported relational bond.

Seven terms were statistically significant in the model predicting therapist-reported relational bond. Specifically, significant effects for individual positive behavior, individual negative behavior, group positive behavior and therapist group management skills were qualified by three significant interactions: individual positive behavior × group positive behavior, individual positive behavior × therapist group management skills, and individual negative behavior × managing deviant talk and behavior. As shown in Figure 3, decomposition of the individual positive behavior × group positive behavior interaction shows that therapist-reported relational bond increases as both individual and group positive behavior scores increase. Moreover, the association between individual positive behavior and higher relational bond scores is stronger at lower levels of group positive behavior (or in other words, child and group positive behavior increase relational bond with the effect of individual behavior greatest when it is inconsistent with the group norm). As shown in Figure 4, decomposition of the individual positive behavior × therapist group management skills interaction shows that individual positive behavior is linked with higher relational bond scores at mean and high levels of group management skills, but not at low levels of group management skills (or in other words, the absence of positive behavior is most detrimental when therapists are high in group management skills). As shown in Figure 5, decomposition of the individual negative behavior × managing deviant talk and behavior interaction shows that more individual negative behavior was linked with lower levels of relational bond at low and mean levels of managing deviant talk and behavior, but not at high levels of managing deviant talk and behavior (or in other words when therapists were better at managing deviant talk and behavior, negative behavior had less impact on the therapists’ perception of the relational bond).

**Figure 6.**
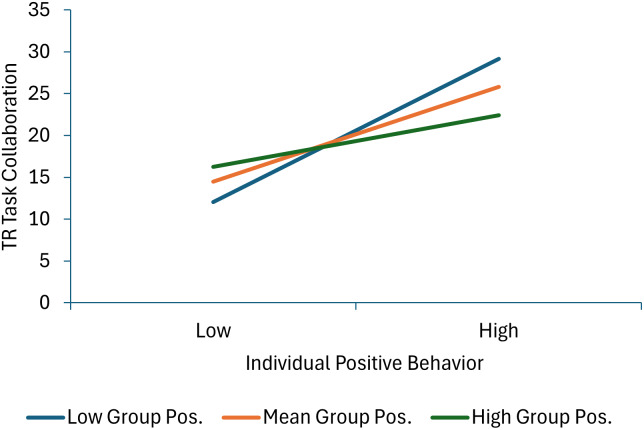
This figure decomposes the interaction between individual and group positive behavior when predicting therapist-reported task collaboration.

**Figure 7.**
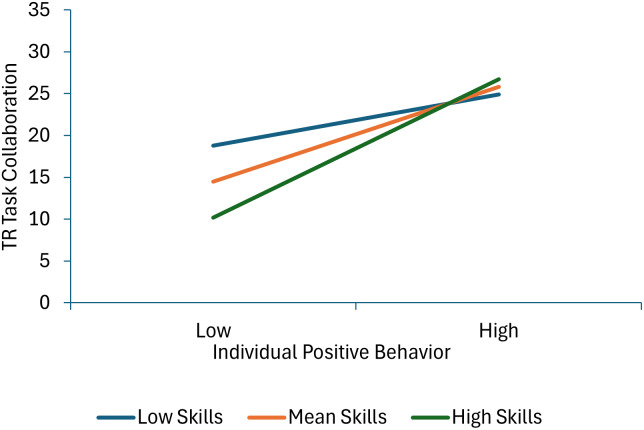
This figure decomposes the interaction between individual positive behavior and group management skills when predicting therapist-reported task collaboration.

Six terms were statistically significant in the model predicting therapist-reported task collaboration. Less individual negative behavior and more therapists’ skill in managing deviant talk and behavior predicated higher task collaboration scores. Effects for individual positive behavior and therapist group management skills were qualified by significant individual positive behavior × group positive behavior and individual positive behavior × therapist group management skills interaction terms. As shown in Figure 6, decomposition of the individual positive behavior × group positive behavior interaction shows that task collaboration increases as individual positive behavior scores increase at low and mean levels of group positive behavior, but not a high levels of group positive behavior (or in other words, child positive behavior increases task collaboration more when it is inconsistent with the group norm). As shown in Figure 7, decomposition of the individual positive behavior × therapist group management skills interaction shows that individual positive behavior is linked with higher task collaboration scores at mean and high levels of group management skills, but not at low levels of therapist group management skills (or in other words, the absence of positive behavior is most detrimental when therapists are high in group management skills).

## Discussion

This study is the first to examine how therapist and child in-session behaviors predict how the TA develops in group-based cognitive-behavioral intervention for children with aggressive behavior problems. TA and in-session behaviors are aspects of the process of intervention and have separately been shown to affect children’s later behavioral outcomes. Thus, exploring the interplay between the alliance development and the behaviors of the individuals involved can potentially provide important implications for the implementation of group intervention. This study found that children’s behavior, both at the individual level as hypothesized (for both positive and negative in-session behavior) and at the group-wide level (for positive behavior only), during the first half of the intervention program, predicted the level of therapist- and child-reported TA that developed over the remainder of the intervention. Therapist behavior also influenced TA, but not in the way anticipated. Although therapists’ clinical skills did not predict TA as hypothesized, therapists’ efforts to manage deviant behavior did predict stronger relational bond and task collaboration. Exploratory analyses indicated that therapist and child in-session behaviors are significantly related to each other, and that children’s in-session behaviors predict TA in different ways, depending on the context of the behavior of the group at large, and on the therapists’ behaviors. These findings contribute to a broader understanding of the developmental pathways that link early behavioral engagement in structured group settings to relational and behavioral adaptation over time.

### Effects of children’s in-session behaviors on the therapeutic alliance

As hypothesized, individual children’s positive and negative in-session behaviors during the first sixteen sessions of the group-based intervention predicted the degree of therapist- and child-rated relational bond and task collaboration. Although the correlations are in the range of small to medium effect sizes, the findings support the validity of the link between child behavior in sessions and subsequent TA. Findings were identical for two different sources of ratings and the two dimensions of TA. Although there are no prior findings about the relation between children’s behaviors and the TA in group work with children with disruptive behaviors, the nature of this behavioral influence on the working alliance is consistent with research with adult clients (Thomas et al., 2005) and child clients (Ovenstad et al., 2020).

Children who show involvement and interest in group activities and who initiate and maintain positive interactions with other group members and with the therapist during the early part of the intervention are likely to perceive that they have established a solid relational bond with the therapist and that they are collaborating in a mutually successful way with the therapist on group tasks. In contrast, children who are acting negatively in the group sessions, by being off-task, inattentive, silly, disruptive, hostile and aggressive, report that they are not engaging well on therapeutic tasks nor developing a good relationship with the therapist. Thus, children’s perception of their relationship with the therapist mirrors the actual behavior they display in sessions.

Interestingly, children’s positive and negative in-session behaviors also relate to the therapist’s perception of the relationship they have developed with the child. When a child engages in substantial amounts of negative behavior in the group, the therapist may perceive that the child is not allied with the therapist or the work at hand, and, in the group context, is disrupting group work and making it much harder for the therapist to develop solid working alliances with other group members. Because a child displaying negative behavior is less likely to acquire emotion regulation and social problem-solving skills from intervention tasks, the child’s ability to relate productively with the therapist does not evolve, and the therapist is likely to continue to have difficulty engaging the child during group sessions. Observable disengaged behavior by children early in therapy should be taken seriously as an indicator of weak alliance development (Ovenstad et al., 2020) and poor outcomes (Lochman, Dishion et al., 2017; Lochman, Boxmeyer et al., 2017).

The direct relation between children’s in-session behaviors and the therapists’ perceptions of the strength of their working alliance is similar to the reciprocity between therapist and child behaviors observed in the therapy sessions. Bivariate correlations show that children engaged in more positive behaviors in sessions had therapists who displayed stronger clinically skilled behaviors, more of the appropriate efforts to structure group sessions and better efforts to manage deviant group behavior. With children who had higher rates of negative behaviors, therapists had lower levels of clinically skilled behaviors and lower rates of group structuring. These associations between child and therapist behaviors likely represent complex bi-directional and reciprocal influences back-and-forth between child and therapist behaviors during sessions, and affect the evolving TA (Nunez et al., 2022).

### Effects of therapists’ in-session behaviors on the therapeutic alliance

#### Therapists’ clinical skills

TA for children with depression (Jungbluth & Shirk, 2009), and with anxiety and trauma-related problems (Crawford et al., 2018; Fjermestad et al., 2021; Hudson et al., 2014; Ovenstad, et al., 2020) has been found to be predicted by clinical behaviors related to flexibility, low levels of critical behavior, and rapport-building, therapist skills which are similar to the clinical skills we examined in this study. However, although the clinical skills assessed in this study have been found to predict children’s behavioral outcomes in the years after the Coping Power intervention ended (Lochman, Dishion et al., 2017), they had no bearing on how the TA manifests within group-based Coping Power.

The failure of these clinical skills to be linked to therapist and child perceptions of the TA in the current study, even though they are related to these children’s longer-term behavioral outcomes (Lochman, Dishion et al., 2017), is likely due in part to the group-based delivery of Coping Power and to the different nature of how alliance relates to the problematic behaviors evident for children in this study. The group therapists’ clinical skills appear to augment the value of the social reinforcement that children receive for their out-of-session behavior, and to provide a model for how to handle difficult and frustrating situations through the leaders’ own behavior in the group which can lead to new schematic models for how children can handle social situations in future years (Lochman, Dishion et al., 2017) but were not primary factors in building the working relationship with the children during sessions. In cognitive-behavioral group therapy, children are relating to their peers as well as the clinician, and successful working relationships in group therapy are likely to involve cohesive multilayered relationships with the peers as well as with the clinician. The therapists’ warmth and flexibility are only part of the network of child and clinician relations that create a bond for the child to the group. In addition, group therapies that are cognitive-behavioral are structured and manualized, thus tending to neutralize, to some extent, the individual bond between a child and the therapist (Alldredge et al., 2021).

### Therapist behaviors which manage deviant behavior

Instead of the clinical skills which have been found to be important for development of TA in individual treatment for internalizing problems, the ways in which clinicians managed deviant behavior in the group sessions did have a direct impact on how both children and clinicians perceived their ability to collaborate on tasks, and children’s perceptions of the relational bond between children and the clinician. The focus here is on how children’s deviant talk and behavior in group sessions can lead to increasing rates of deviant behavior by the peer group. Therapists who redirected group attention away from deviant talk and towards group activities and tasks, *and* avoiding encouraging deviant talk themselves through their own positive attention and comments about the deviant statements, perceived that they had also developed more productive abilities to work in collaborative ways on group tasks with their child clients. Surprisingly, the clinicians’ ability to manage deviant behavior also has important effects on children’s perceptions of task collaboration, in line with the therapists’ perceptions, and even influenced children’s perceptions of the strength of the relational bond with the therapist.

The clinician effectively manages deviant talk or behavior by stating that the deviant behavior or talk that was exhibited was not appropriate for the group’s work, then quicky and seamlessly redirects the group back to an earlier non-deviant discussion point from another group member or redirects to the current topic and activity (e.g. organization and study skills). The clinician avoids giving positive attention and support to the deviant talk while they are redirecting back to the task. As seen in Supplementary Table 2, managing deviant behavior was significantly correlated with the other therapist behavior codes, indicating that therapists who provided redirection for instances of deviant talk and behavior also, throughout the session, responded in warm, flexible ways, and provided clear structure for activities and consequences for problem behaviors. Therapists who do not manage deviant behaviors well are likely not structuring the sessions well and are likely to be relatively rigid and lack warmth in their interactions with the children, but it is their weaker ability to manage deviant behavior that is most linked to the development of TA. If a clinician provided consequences for the deviant behavior but did not redirect back to the task quicky, results indicate that the clinician would be less able to develop a strong therapeutic alliance. Providing consequences is important for changing children’s behaviors, but in the absence of efficient redirection to the task, clinician provided consequences will not enhance the therapeutic alliance. A likely optimal clinical strategy would be for the clinician to redirect the group to the task at hand (to enhance therapeutic alliance) and then to later provide consequences as needed to the individual who exhibited the deviant behavior (to promote behavior change). If the group has two co-leaders, then one can provide the redirection to task for the rest of the group members, and the second co-leader can provide consequences to the child who engaged in the deviant talk. In summary, the therapists typically can re-establish appropriate norms by directly stating that the deviant behavior or comment does not fit with the constructive task at hand or with general expectations for the group. The re-direction places positive value on task-relevant talk and de-values deviant talk and behavior.

These efforts by Coping Power therapists to manage deviant behavior in the group are consistent with recommendations for group therapists to be directive and provide sufficient structure and containment for group discussions (Haen & Aronson, 2024), and to redirect and cut-off hostile and negative talk and behaviors (Gladding, 2003). Adolescent clients themselves also see the value of therapist redirection, as evident in a qualitative study of adolescent perceptions of group therapist behaviors (Pingitore & Ferszt, 2017). Redirection has been recognized as an important skill for adults to use with children in other group settings as well. Teachers who create safe and conducive learning environments set clear behavioral expectations (McDaniel et al., 2024) and use brief clear redirects for inappropriate student behaviors (Solomon et al., 2000).

### Effects of children’s in-session behavior in the context of peer and clinician behavior

#### How peer behavior in group moderates the effect of children’s in-session behavior

Following Ryan et al.’s (2023) suggestion, this study provided a unique opportunity to examine how group level behaviors may affect how child behavior was related to TA. Individual × group behavior interactions have long been considered in the study of how youth are perceived by peers. Many studies find that aggressive behavior is less associated with peer dislike when aggression is more common in the peer group, suggesting that behavior norms affect how the behavior of an individual child is viewed by peers (e.g., Barth et al., 2004). However, when it comes to how adult therapists and clients may perceive alliance based on the group context, much is still unknown. Thus, it was important to explore how a child’s behavior in the group may affect relationships with group therapists differently depending on how the group behaved collectively.

Results indicated several instances in which the group’s behavior moderated how the child’s behavior was related to relational bond and task collaboration. First, child negative behavior was less strongly related to *child report* of relational bond as group negative behavior decreased. It may be that when groups are harder to manage for therapists, a child’s negative behavior is detrimental to bonding with the child. It may also be that in a group with more deviant behavior a child who aligns with the group norm is more oriented to peer approval and cares less about alliance with the adult therapist. However, when negative behavior is less common, therapists may be better able to connect with individual children even when they are misbehaving in the group and the child may perceive their relationship with the therapist more positively.

We also found interactions for how therapists reported TA. Child positive behavior was more strongly positively associated with therapist-reported relational bonds and collaborative alliance when groups were lower on positive behavior. In this case, a child who is high in positive behavior, but in a group who is not, may stand out more to a therapist. In this scenario, the therapist may perceive the positive child behavior to indicate more bond between themself and the child.

These findings support the notion of a multilevel developmental model in which child outcomes are shaped not only by individual characteristics but also by broader group norms and the intervention ecology. In line with ecological models of development, peer behavior moderated the link between child conduct and perceived alliance, emphasizing that therapeutic processes are embedded within social-contextual dynamics. Understanding the reciprocal influences between child behavior, peer norms, and adult responses is essential to designing developmentally sensitive group interventions.

#### How therapist behavior moderates the effect of children’s in-session behavior

Our findings suggest nuance in the relation between children’s behavior in group sessions and TA. Despite the overall main effect finding that children’s negative behaviors reduced TA, child negative behavior did not affect child or therapist report of relational bond when therapists had high levels of managing deviant behavior. That is, children who displayed off-task and disruptive behaviors during sessions could maintain positive feelings about their relationship with the group therapist, if the therapist managed the problem behavior effectively. These findings are reminiscent of well-established concepts in the parenting and education literature, namely, authoritative parenting and “warm demanders” in the classroom (Bondy & Ross, 2008). For both, adult influence is optimized under conditions of firm limits, warmth, and emotionally regulated responses. Managing deviant behavior in this study involved affectively neutral redirection to task, underscoring the therapists’ expectations for appropriate behavior along with their investment in children’s learning. In educational settings, children report positive feelings about teachers who maintain orderly classrooms conducive to learning (García-Moya, et al., 2020), and this relation holds for children who display challenging behaviors at school (Gibbs et al., 2022). In sum, children who display challenging behaviors in group settings can appreciate the need for redirection and can maintain positive attitudes about their adult therapists when management is respectful, consistent, and communicates concern for the children’s best interests.

For children who displayed low levels of positive behavior during group sessions, there was an interaction between perceived relational bond and therapists’ group management skill. When therapists were actively working to enforce rules and expectations, reinforce appropriate behavior, and engage children’s participation, they perceived children who were positive as higher in relational bond, while perceiving children who were less positive as less bonded. In contrast, therapists who were less skilled in the use of group management did not vary in their ratings of relational bond based on children’s degree of positive behavior. Therapists who have lower group management abilities may feel overwhelmed, resulting in them trying to focus on being more organized and efficient in providing the content and guiding the group’s behavioral expectations, and perhaps leading them to not notice individual differences in children’s behaviors. In a related vein, therapists who have stronger group management abilities should be cautious about having expectations that relatively neutral child in-session behavior necessarily indicates that the child has no interest in developing a therapeutic relationship and instead should further seek to proactively develop the bond. Therapists received initial training and weekly feedback about in-group behavior management strategies and may have developed expectations about how using these strategies would affect children’s experience of the program. Therapists therefore may have seen positive child behaviors in this context as an indication that the child was invested in gaining the therapist’s approval, while low levels of positive behavior could be seen as disinterest in the therapist and lack of connection.

A similar pattern emerged for therapist ratings of task collaboration when children were low in positive behavior and therapists were high on group management skill. Again, therapists who carefully attended to and implemented the program’s behavior management procedures might have been primed to draw connections between these strategies and children’s degree of positive behavior. Children who were not very positive may have been viewed by therapists as non-responsive to external support and lacking in internal motivation, resulting in lower ratings on task collaboration. In line with these interpretations, Ormhaug et al. (2015) suggested that therapists tend to judge alliance based on youths’ ability to collaborate on therapeutic activities.

### Limitations

Several limitations of this study warrant consideration. First, the analyses were conducted using secondary data from an existing trial, limiting the ability to draw causal links between in-session behaviors and TA. Second, the study focused on a group modality of Coping Power for children with externalizing behavior problems and findings may not generalize to other therapeutic modalities. Third, although alliance was assessed at the middle and end of treatment, it was difficult to capture temporal dynamics of alliance formation and real-time changes in alliance across sessions could offer additional insight. Fourth, results may be underestimating the extent to which individual behavior and group behavior or child and therapist behavior interact to predict TA because our analyses were likely underpowered to detect interaction effects (McClelland & Judd, 1993). Fifth, alliance ratings relied on child and therapist reports, which may be influenced by social desirability or differing perceptions of alliance strength, and the lower internal consistency for child-reported TA may account for overall weaker findings predicting child-reported TA. Finally, while the study incorporated observed child and therapist behaviors, the observational coding system may not have fully captured the nuances of interactional dynamics, especially given the complex and reciprocal nature of group sessions.

## Conclusions

This study provides novel evidence that both child and therapist in-session behaviors contribute to the development of the TA in group-based cognitive-behavioral intervention for youth with externalizing problems. Specifically, children’s early positive engagement and the therapist’s skill in managing deviant behavior were consistent predictors of stronger therapeutic relationships. These findings underscore the interactive and contextual nature of alliance-building, particularly within the group therapy setting where multiple relational dynamics are at play. The results also challenge assumptions that therapist warmth and flexibility are sufficient to build alliance with challenging youth, emphasizing instead the critical role of structured behavior management in fostering engagement and connection.

Future research should build on these findings by addressing several key areas. First, longitudinal, session-by-session assessments of TA would offer a more nuanced understanding of its development and fluctuation over time. Second, studies should explore how alliance functions differently across diverse treatment formats and populations, including youth with internalizing symptoms and neurodevelopmental conditions. Third, future work should use multi-method and multi-informant approaches, incorporating observational measures of alliance alongside self-report, to reduce potential bias. Fourth, our findings suggest need for further examination of specific therapist training or supervision strategies that enhance behavior management skills in group settings, as these appear to be more influential than traditionally emphasized clinical skills. Finally, research should further investigate group-level processes, including peer influences and emergent group norms, to better understand how collective behavior shapes individual alliance development and treatment outcomes.

## Supporting information

10.1017/S0954579425101119.sm001 Laird et al. supplementary material Laird et al. supplementary material

## Data Availability

Data and code are available on the Open Science Framework: https://doi.org/10.17605/OSF.IO/SY8Z6. Materials may be obtained by emailing the authors.

## Supplementary material

The supplementary material for this article can be found at https://doi.org/10.1017/S0954579425101119.

## Pre-registration statement

The research described in the article was not preregistered because the research was a secondary analysis that required some exploratory analyses. The rationale and all exploratory analyses are documented in the manuscript and supplementary material.
