# Supplementary material for: What builds the bond? Child and therapist behavior in a group intervention for aggression
Source: Dev Psychopathol. 2026 Jan 23:1–13. Online ahead of print. doi: 10.1017/S0954579425101119 (PMC12888082; doi:10.1017/S0954579425101119)
Supplement: Laird et al. supplementary material [file S0954579425101119sup001.docx]

**Multi-level Model Equation**

The focal model included seven primary predictors and eight interaction terms. Specifically, TA was predicted by individual positive behavior (IP), individual negative behavior (IN), group positive behavior (GP), group negative behavior (GN), therapist group management skills (TG), therapist clinical skills (TC), and therapist skill at managing deviant talk and behavior (TD) and the following interaction terms: individual positive behavior × group positive behavior, individual negative behavior × group negative behavior, individual positive behavior × therapist group management skills, individual positive behavior × therapist clinical skills, individual positive behavior × therapist managing deviant talk and behavior, individual negative behavior × therapist group management skills, individual negative behavior × therapist clinical skills, individual negative behavior × therapist managing deviant talk and behavior.

Level 1(individual) Equation*: TA_ij_* = *β*_0J_ + *β*_1_IP_ij_ + *β*_2_IN_ij_ + *e_ij_*

Level 2 (Group) Equation2: β_0j_= γ_00_ + γ_01_GP_j_ + γ_02_GN_j_ + γ_03_TG_j_ + γ_04_TC_j_ + γ_05_TD_j_ + μ_0j_

*β*_1 =_ γ_10_ + γ_11_GP_j_ + γ_12_TG_j_ + γ_13_TC_j_ + γ_14_TD_j_

*β*_2 =_ γ_20_ + γ_21_GN_j_ + γ_22_TG_j_ + γ_23_TC_j_ + γ_24_TD_j_

Combined Equation: *TA_ij_* = γ_00_ + γ_01_GP_j_ + γ_02_GN_j_ + γ_03_TG_j_ + γ_04_TC_j_ + γ_05_TD_j_ + γ_10_IP_ij_ + γ_11_IP_ij_ × GP_j_ + γ_12_IP_ij_ × TG_j_ + γ_13_IP_ij_ × TC_j_ + γ_14_IP_ij_ ×TD_j_ + γ_20_IN_ij_ + γ_21_IN_ij_ × GN_j_ + γ_22_IN_ij_ × TG_j_ + γ_23_IN_ij_ × TC_j_ + γ_24_IN_ij_ × TD_j_ + μ_0j_ + *e_ij_*

Parameter estimates provide evidence that TA is linked with individual positive behavior (γ_10_), individual negative behavior (γ_20_), group positive behavior (γ_01_), group negative behavior (γ_02_), therapist group management skills (γ_03_), therapist clinical skills (γ_04_), and therapist skill at managing deviant talk and behavior (γ_05_). Parameter estimates for the eight interaction terms test whether TA is predicted by the individual positive behavior × group positive behavior interaction (γ_11_), the individual negative behavior × group negative behavior interaction (γ_21_), the individual positive behavior × therapist group management skills interaction (γ_12_), the individual positive behavior × therapist clinical skills interaction (γ_13_), the individual positive behavior × therapist managing deviant talk and behavior interaction (γ_14_), the individual negative behavior × therapist group management skills interaction (γ_22_), the individual negative behavior × therapist clinical skills interaction (γ_23_), and the individual negative behavior × therapist managing deviant talk and behavior interaction (γ_24_).

**Supplementary Table 1**: *Intra-class Correlations for Child and Therapist Behavior*

|  | Individual Child Behavior | | Therapist Behavior | | |
| --- | --- | --- | --- | --- | --- |
| Level | Positive Behavior | Negative Behavior | Group Management Skills | Clinical Skills | Managing Deviant Talk & Behavior |
| Within Sessions | .011 | .004 | .010 | .000 | .001 |
| Across Sessions | .024 | .029 | .056 | .028 | .001 |
| Across Groups | .192 | .123 | .323 | .288 | .024 |
| Across Children | .235 | .179 | -- | -- | -- |

ICCS values indicate the proportion of variance estimated to lie at each level.

**Supplementary Table 2:** *Bivariate Correlations among all Study Variables*

|  | 1 | 2 | 3 | 4 | 5 | 6 | 7 | 8 | 9 | 10 |
| --- | --- | --- | --- | --- | --- | --- | --- | --- | --- | --- |
| 1. CR Relational Bond |  |  |  |  |  |  |  |  |  |  |
| 2. CR Task Collaboration | .73^***^ |  |  |  |  |  |  |  |  |  |
| 3. TR Relational Bond | .48^***^ | .35^***^ |  |  |  |  |  |  |  |  |
| 4. TR Task Collaboration | .49^***^ | .35^***^ | .81^***^ |  |  |  |  |  |  |  |
| 5. Individual Positive Behavior | .24^**^ | .20^*^ | .39^**^ | .39^***^ |  |  |  |  |  |  |
| 6. Individual Negative Behavior | -.17* | -.16* | -.30^***^ | -.22^**^ | -.27^***^ |  |  |  |  |  |
| 7. Group Positive Behavior | .20^*^ | .18^*^ | .16^*^ | .19^*^ | .84^***^ | -.22^**^ |  |  |  |  |
| 8. Group Negative Behavior | -.06 | -.11 | -.09 | .00 | -.23^**^ | .80^***^ | -.27^***^ |  |  |  |
| 9. Group Management Skills | .03 | .13 | -.04 | -.13 | .51^***^ | -.40^***^ | .61^***^ | -.50^***^ |  |  |
| 10.Clinical Skills | .05 | .15 | -.00 | -.07 | .33^***^ | -.32^***^ | .39^***^ | -.40^***^ | .63^***^ |  |
| 11. Manage Deviant Talk/Behavior | .17^*^ | .20^*^ | .12 | .18^*^ | .39^***^ | .07 | .46^***^ | .08 | .37^***^ | .28^***^ |
| *Note.* *N*s = 146-180. * *p* < .05, ***p*<.01, ****p*<.001 | | | | | | | | | | |

**Supplementary Table 3**: Predictors Tested Individually

***Table 3A****: Child-Reported Therapeutic Alliance Predicted by Child and Therapist Behavior*

|  | Relational Bond | | | | | Therapeutic Alliance | | | | |
| --- | --- | --- | --- | --- | --- | --- | --- | --- | --- | --- |
| Predictor | β | *s.e.* | *df* | *p* | 95% CI | β | *s.e.* | *df* | *p* | 95% CI |
| Individual Positive (IP) | .381 | .136 | 56.803 | .007 | [.109, .654] | .278 | .123 | 61.151 | .028 | [.032, .524] |
| Individual Negative (IN) | -.666 | .298 | 78.537 | .028 | [-1.259, -.073] | -.513 | .264 | 80.382 | .056 | [-1.039, .013] |
| Group Positive (GP) | .353 | .169 | 23.947 | .048 | [.004, .702] | .283 | .150 | 28.213 | .07 | [-.024, .589] |
| Group Negative (GN) | -.211 | .423 | 27.343 | .621 | [-1.078, .655] | -.389 | .365 | 32.576 | .293 | [-1.132, .353] |
| Group Management Skills (GM) | .044 | .096 | 24.474 | .653 | [-.155, .242] | .113 | .082 | 28.964 | .175 | [-.053, .280] |
| Clinical Skills (CS) | .194 | .344 | 25.381 | .578 | [-.515, .902] | .448 | .289 | 29.705 | .132 | [-.143, 1.039] |
| Managing Deviant Talk & Behavior (MD) | 1.661 | .949 | 22.721 | .093 | [-.303, 3.625] | 1.725 | .805 | 26.428 | .041 | [.072, 3.378] |

Note: Predicted variables were tested individually in a series of 7 models for each outcome variable.

***Table 3B****: Therapist-Reported Therapeutic Alliance Predicted by Child and Therapist Behavior*

|  | Relational Bond | | | | | Task Collaboration | | | | |
| --- | --- | --- | --- | --- | --- | --- | --- | --- | --- | --- |
| Predictor | β | *s.e.* | *df* | *p* | 95% CI | β | *s.e.* | *df* | *p* | 95% CI |
| Individual Positive (IP) | .797 | .140 | 57.026 | <.001 | [.517, 1.077] | .789 | .139 | 75.998 | <.001 | [.513, 1.066] |
| Individual Negative (IN) | -1.338 | .298 | 89.469 | <.001 | [-1.93, -.745] | -1.398 | .302 | 124.938 | <.001 | [-1.996, -.801] |
| Group Positive (GP) | .288 | .163 | 23.606 | .091 | [-.049, .626] | .333 | .179 | 23.776 | .075 | [-.036, .702] |
| Group Negative (GN) | -.348 | .389 | 26.477 | .379 | [-1.147, .451] | -.003 | .431 | 25.845 | .995 | [-.889, .884] |
| Group Management Skills (GM) | -.045 | .092 | 25.334 | .632 | [-.234, .145] | -.118 | .098 | 24.524 | .241 | [-.321, .084] |
| Clinical Skills (CS) | -.019 | .328 | 25.395 | .954 | [-.694, .656] | -.206 | .356 | 24.693 | .568 | [-.941, .528] |
| Managing Deviant Talk & Behavior (MD) | 1.195 | .936 | 25.199 | .213 | [-.731, 3.121] | 1.72 | 1.001 | 24.833 | .098 | [-.342, 3.783] |

Note: Predicted variables were tested individually in a series of 7 models for each outcome variable.

**Supplementary Table 4**: Mid-point, Endpoint, and Endpoint controlling for mid-point

***Table 4A****: Child-Reported Therapeutic Alliance at* ***Mid-point*** *Predicted by Child and Therapist Behavior*

|  | Relational Bond | | | | | Task Collaboration | | | | |  |
| --- | --- | --- | --- | --- | --- | --- | --- | --- | --- | --- | --- |
| Predictor | Β | *s.e.* | *df* | *p* | 95% CI | β | *s.e.* | *df* | *p* | 95% CI | |
| Individual Positive (IP) | .226 | .286 | 43.504 | .333 | [-.339, .791] | .272 | .261 | 132.897 | .299 | [-.245, .789] | |
| Individual Negative (IN) | -1.558 | .601 | 38.320 | .665 | [-2.746, -.370] | -1.221 | .548 | 133.588 | .027 | [-2.304, -.138] | |
| Group Positive (GP) | .451 | .461 | 21.302 | .041 | [-.478, 1.38] | .153 | .377 | 57.402 | .685 | [-.601, .908] | |
| Group Negative (GN) | .391 | .895 | 18.604 | .720 | [-1.421, 2.203] | -.316 | .726 | 53.199 | .665 | [-1.771, 1.139] | |
| Group Management Skills (GM) | -.499 | .229 | 21.246 | .049 | [-.974, -.024] | -.284 | .174 | 24.909 | .115 | [-.643, .074 | |
| Clinical Skills (CS) | .226 | .620 | 76.506 | .892 | [-1.075, 1.526] | .215 | .465 | 21.926 | .648 | [-.749, 1.179] | |
| Managing Deviant Talk & Behavior (MD) | 3.908 | 1.872 | 86.418 | .045 | [.018, 7.797] | 4.698 | 1.421 | 24.518 | .003 | [1.768, 7.629] | |
| IP X GP | -.022 | .163 | 119.812 | .739 | [-.347, .302] | -.108 | .136 | 59.589 | .430 | [-.381, .164] | |
| IN X GN | -1.222 | .601 | 68.866 | .966 | [-2.417, -.026] | -.804 | .512 | 80.777 | .120 | [-1.823, .215] | |
| IP X GM | .028 | .084 | 45.844 | .285 | [-.139, .195] | .015 | .074 | 105.278 | .842 | [-.132, .161] | |
| IP X CS | -.012 | .287 | 98.372 | .101 | [-.585, .560] | -.083 | .239 | 56.436 | .729 | [-.561, .395] | |
| IP X MD | -.756 | .699 | 131.787 | .292 | [-2.164, .652] | -.375 | .564 | 39.632 | .510 | [-1.515, .764] | |
| IN X GM | -.314 | .190 | 129.818 | .009 | [-.691, .062] | -.277 | .163 | 83.489 | .093 | [-.601, .047] | |
| IN X CS | -.582 | .550 | 43.504 | .333 | [-1.670, .507] | -.685 | .490 | 122.081 | .165 | [-1.655, .285] | |
| IN X MD | 5.263 | 1.994 | 38.320 | .665 | [1.319, 9.207] | 3.597 | 1.767 | 116.429 | .044 | [.097, 7.097] | |

***Table 4B****: Therapist-Reported Therapeutic Alliance at* ***Mid-point*** *Predicted by Child and Therapist Behavior*

|  | Relational Bond | | | | | Task Collaboration | | | | |
| --- | --- | --- | --- | --- | --- | --- | --- | --- | --- | --- |
| Predictor | β | *s.e.* | *df* | *p* | 95% CI | β | *s.e.* | *df* | *p* | 95% CI |
| Individual Positive (IP) | 1.059 | .216 | 132.614 | <.001 | [.633, 1.486] | .989 | .219 | 133.929 | <.001 | [.557, 1.422] |
| Individual Negative (IN) | -1.852 | .447 | 130.736 | <.001 | [-2.736, -.967] | -1.947 | .450 | 131.343 | <.001 | [-2.837, -1.057] |
| Group Positive (GP) | -.545 | .315 | 40.436 | .092 | [-1.182, .092] | -.352 | .308 | 53.931 | .259 | [-.970, .266] |
| Group Negative (GN) | .654 | .558 | 31.811 | .250 | [-.484, 1.791] | .808 | .543 | 44.771 | .144 | [-.286, 1.901] |
| Group Management Skills (GM) | -.501 | .144 | 14.797 | .003 | [-.809, -.194] | -.458 | .137 | 19.549 | .003 | [-.744, -.173] |
| Clinical Skills (CS) | .083 | .376 | 11.782 | .828 | [-.738, .905] | -.039 | .353 | 15.649 | .913 | [-.790, .711] |
| Managing Deviant Talk & Behavior (MD) | 1.687 | 1.176 | 14.743 | .173 | [-.825, 4.198] | 2.527 | 1.115 | 19.526 | .035 | [.198, 4.857] |
| IP X GP | -.158 | .111 | 42.207 | .161 | [-.381, .066] | -.196 | .108 | 46.117 | .076 | [-.413, .022] |
| IN X GN | -.568 | .419 | 69.150 | .180 | [-1.404, .269] | -.519 | .414 | 75.974 | .215 | [-1.344, .307] |
| IP X GM | .177 | .062 | 95.630 | .005 | [.054, .299] | .102 | .062 | 96.979 | .101 | [-.020, .224] |
| IP X CS | -.130 | .192 | 35.393 | .502 | [-.520, .259] | -.061 | .187 | 39.770 | .746 | [-.438, .316] |
| IP X MD | -.088 | .465 | 25.038 | .852 | [-1.045, .870] | .015 | .448 | 29.629 | .974 | [-.900, .929] |
| IN X GM | -.211 | .135 | 64.157 | .123 | [-.481, .058] | -.175 | .133 | 68.666 | .192 | [-.441, .090] |
| IN X CS | .373 | .402 | 111.473 | .355 | [-.422, 1.169] | -.245 | .401 | 109.438 | .542 | [-1.041, .550] |
| IN X MD | 3.686 | 1.473 | 102.405 | .014 | [.764, 6.607] | .884 | 1.468 | 100.997 | .548 | [-2.029, 3.797] |

***Table 4C****: Child-Reported Therapeutic Alliance at* ***Endpoint*** *Predicted by Child and Therapist Behavior*

|  | Relational Bond | | | | | Task Collaboration | | | | |
| --- | --- | --- | --- | --- | --- | --- | --- | --- | --- | --- |
| Predictor | β | *s.e.* | *df* | *p* | 95% CI | β | *s.e.* | *df* | *p* | 95% CI |
| Individual Positive (IP) | .260 | .280 | 134.886 | .354 | [-.293, .813] | .307 | .276 | 135.239 | .269 | [-.240, .853] |
| Individual Negative (IN) | -.712 | .581 | 135.532 | .222 | [-1.860, .436] | -.582 | .574 | 135.614 | .312 | [-1.716, .552] |
| Group Positive (GP) | .246 | .389 | 53.480 | .529 | [-.533, 1.025] | -.016 | .376 | 62.389 | .967 | [-.767, .736] |
| Group Negative (GN) | .766 | .742 | 49.682 | .307 | [-.724, 2.256] | .237 | .716 | 59.033 | .742 | [-1.197, 1.67] |
| Group Management Skills (GM) | -.145 | .174 | 20.687 | .414 | [-.507, .217] | .003 | .165 | 24.367 | .987 | [-.338, .343] |
| Clinical Skills (CS) | -.316 | .467 | 19.100 | .506 | [-1.294, .661] | .072 | .443 | 22.783 | .873 | [-.845, .988] |
| Managing Deviant Talk & Behavior (MD) | 2.172 | 1.389 | 18.943 | .134 | [-.735, 5.08] | 1.384 | 1.315 | 22.224 | .304 | [-1.342, 4.11] |
| IP X GP | .005 | .152 | 42.803 | .974 | [-.302, .312] | -.083 | .146 | 46.238 | .575 | [-.377, .212] |
| IN X GN | -1.083 | .532 | 73.061 | .045 | [-2.143, -.022] | -.019 | .517 | 77.940 | .971 | [-1.048, 1.01] |
| IP X GM | .050 | .078 | 96.949 | .522 | [-.104, .204] | .099 | .076 | 98.171 | .192 | [-.051, .25] |
| IP X CS | -.272 | .261 | 45.880 | .303 | [-.797, .253] | -.182 | .252 | 50.053 | .473 | [-.687, .323] |
| IP X MD | -.269 | .590 | 29.570 | .652 | [-1.476, .937] | -.644 | .564 | 32.706 | .262 | [-1.793, .504] |
| IN X GM | -.164 | .168 | 71.263 | .332 | [-.498, .171] | -.120 | .163 | 73.975 | .464 | [-.444, .205] |
| IN X CS | -.719 | .522 | 115.457 | .171 | [-1.753, .315] | .143 | .511 | 114.622 | .780 | [-.870, 1.156] |
| IN X MD | 4.384 | 1.823 | 106.314 | .018 | [.770, 7.999] | 1.140 | 1.782 | 105.844 | .524 | [-2.394, 4.674] |

**Table 4D**: *Therapist-Reported Therapeutic Alliance at* ***Endpoint*** *Predicted by Child and Therapist Behavior*

|  | Relational Bond | | | | | Task Collaboration | | | | |
| --- | --- | --- | --- | --- | --- | --- | --- | --- | --- | --- |
| Predictor | β | *s.e.* | *df* | *p* | 95% CI | β | *s.e.* | *df* | *p* | 95% CI |
| Individual Positive (IP) | .947 | .246 | 113.812 | <.001 | [.459, 1.435] | .964 | .249 | 113.916 | <.001 | [.471, 1.458] |
| Individual Negative (IN) | -2.589 | .542 | 114 | <.001 | [-3.663, -1.515] | -2.629 | .548 | 114 | <.001 | [-3.714, -1.544] |
| Group Positive (GP) | -.169 | .404 | 28.367 | .679 | [-.997, .658] | -.431 | .389 | 31.746 | .277 | [-1.224, .362] |
| Group Negative (GN) | .347 | .767 | 31.301 | .654 | [-1.216, 1.911] | .445 | .741 | 36.034 | .552 | [-1.058, 1.948] |
| Group Management Skills (GM) | -.701 | .195 | 14.274 | .003 | [-1.118, -.283] | -.663 | .183 | 15.139 | .002 | [-1.053, -.274] |
| Clinical Skills (CS) | .051 | .540 | 14.296 | .926 | [-1.105, 1.208] | .102 | .507 | 15.240 | .843 | [-.977, 1.18] |
| Managing Deviant Talk & Behavior (MD) | 2.354 | 1.769 | 16.589 | .201 | [-1.386, 6.094] | 4.275 | 1.669 | 17.521 | .020 | [.761, 7.79] |
| IP X GP | -.320 | .137 | 57.622 | .023 | [-.595, -.046] | -.259 | .134 | 51.698 | .059 | [-.528, .011] |
| IN X GN | .304 | .510 | 63.296 | .553 | [-.715, 1.324] | -.208 | .502 | 60.605 | .680 | [-1.212, .796] |
| IP X GM | .169 | .069 | 102.976 | .017 | [.031, .307] | .132 | .069 | 98.464 | .060 | [-.006, .27] |
| IP X CS | .189 | .240 | 53.527 | .435 | [-.292, .669] | -.064 | .235 | 48.641 | .787 | [-.535, .408] |
| IP X MD | .456 | .616 | 35.852 | .464 | [-.794, 1.705] | .539 | .596 | 33.781 | .372 | [-.672, 1.75] |
| IN X GM | -.090 | .155 | 89.576 | .560 | [-.397, .217] | -.313 | .154 | 85.931 | .045 | [-.618, -.008] |
| IN X CS | .493 | .475 | 112.764 | .302 | [-.449, 1.434] | .157 | .481 | 113.621 | .745 | [-.796, 1.109] |
| IN X MD | 4.053 | 1.799 | 108.221 | .026 | [.487, 7.618] | 3.648 | 1.827 | 110.655 | .048 | [.027, 7.268] |

***Table 4E****: Child-Reported Therapeutic Alliance at* ***Endpoint (controlling for Mid-point)*** *Predicted by Child and Therapist Behavior*

|  | Relational Bond | | | | | Task Collaboration | | | | |
| --- | --- | --- | --- | --- | --- | --- | --- | --- | --- | --- |
| Predictor | β | *s.e.* | *df* | *p* | 95% CI | β | *s.e.* | *df* | *p* | 95% CI |
| TA at Mid-point | .439 | .071 | 132 | <.001 | [.299, .580] | .469 | .080 | 132 | <.001 | [.310, .628] |
| Individual Positive (IP) | .155 | .251 | 132 | .538 | [-.341, .652] | .155 | .252 | 132 | .539 | [-.344, .654] |
| Individual Negative (IN) | -.025 | .529 | 132 | .963 | [-1.072, 1.022] | -.004 | .528 | 132 | .994 | [-1.049, 1.041] |
| Group Positive (GP) | .159 | .317 | 132 | .617 | [-.468, .787] | -.017 | .319 | 132 | .959 | [-.647, .614] |
| Group Negative (GN) | .393 | .611 | 132 | .521 | [-.815, 1.602] | .261 | .614 | 132 | .672 | [-.954, 1.476] |
| Group Management Skills (GM) | .006 | .135 | 132 | .962 | [-.260, .273] | .123 | .134 | 132 | .361 | [-.142, .388] |
| Clinical Skills (CS) | -.513 | .348 | 132 | .142 | [-1.201, .174] | -.072 | .349 | 132 | .837 | [-.763, .619] |
| Managing Deviant Talk & Behavior (MD) | .491 | 1.105 | 132 | .657 | [-1.694, 2.676] | -1.103 | 1.139 | 132 | .335 | [-3.357, 1.15] |
| IP X GP | -.064 | .122 | 132 | .601 | [-.304, .177] | -.067 | .122 | 132 | .584 | [-.309, .175] |
| IN X GN | -.703 | .446 | 132 | .117 | [-1.584, .179] | .376 | .446 | 132 | .402 | [-.507, 1.259] |
| IP X GM | .054 | .065 | 132 | .409 | [-.075, .182] | .117 | .065 | 132 | .075 | [-.012, .246] |
| IP X CS | -.183 | .208 | 132 | .380 | [-.595, .228] | -.156 | .209 | 132 | .458 | [-.569, .257] |
| IP X MD | .363 | .460 | 132 | .431 | [-.547, 1.274] | -.392 | .458 | 132 | .394 | [-1.298, .514] |
| IN X GM | -.050 | .139 | 132 | .723 | [-.325, .226] | .062 | .141 | 132 | .661 | [-.217, .341] |
| IN X CS | -.455 | .451 | 132 | .316 | [-1.347, .438] | .341 | .451 | 132 | .451 | [-.552, 1.234] |
| IN X MD | 2.807 | 1.581 | 132 | .078 | [-.321, 5.935] | -.772 | 1.595 | 132 | .629 | [-3.928, 2.384] |

Note: Models failed to converge.

***Table 4F****: Therapist-Reported Therapeutic Alliance at* ***Endpoint (controlling for Mid-point)*** *Predicted by Child and Therapist Behavior*

|  | Relational Bond | | | | | Task Collaboration | | | | |
| --- | --- | --- | --- | --- | --- | --- | --- | --- | --- | --- |
| Predictor | β | *s.e.* | *df* | *p* | 95% CI | β | *s.e.* | *df* | *p* | 95% CI |
| TA at Mid-point | .738 | .073 | 111.290 | <.001 | [.594, .882] | .434 | .094 | 112.969 | <.001 | [.249, .62] |
| Individual Positive (IP) | .249 | .192 | 110.691 | .197 | [-.131, .630] | .590 | .245 | 111.853 | .018 | [.104, 1.076] |
| Individual Negative (IN) | -.651 | .438 | 112.999 | .140 | [-1.519, .217] | -1.458 | .561 | 112.952 | .011 | [-2.569, -.347] |
| Group Positive (GP) | .137 | .295 | 32.756 | .645 | [-.463, .738] | -.313 | .340 | 36.073 | .364 | [-1.002, .377] |
| Group Negative (GN) | -.595 | .565 | 36.210 | .299 | [-1.740, .55] | -.178 | .661 | 42.922 | .789 | [-1.512, 1.156] |
| Group Management Skills (GM) | -.266 | .148 | 18.741 | .088 | [-.576, .044] | -.441 | .160 | 17.471 | .013 | [-.778, -.103] |
| Clinical Skills (CS) | .050 | .392 | 16.224 | .900 | [-.780, .88] | .146 | .425 | 15.680 | .736 | [-.756, 1.048] |
| Managing Deviant Talk & Behavior (MD) | 1.484 | 1.287 | 18.798 | .263 | [-1.211, 4.179] | 3.235 | 1.427 | 18.804 | .035 | [.247, 6.223] |
| IP X GP | -.195 | .100 | 58.956 | .056 | [-.396, .006] | -.143 | .120 | 42.010 | .238 | [-.385, .098] |
| IN X GN | .839 | .374 | 66.036 | .028 | [.091, 1.587] | .096 | .450 | 54.896 | .831 | [-.806, .999] |
| IP X GM | .044 | .052 | 102.851 | .396 | [-.059, .147] | .092 | .063 | 86.773 | .150 | [-.034, .219] |
| IP X CS | .262 | .174 | 58.554 | .138 | [-.087, .611] | -.088 | .206 | 41.940 | .671 | [-.505, .328] |
| IP X MD | .207 | .448 | 39.341 | .646 | [-.699, 1.113] | .267 | .518 | 29.730 | .611 | [-.792, 1.325] |
| IN X GM | .143 | .115 | 90.626 | .217 | [-.085, .37] | -.197 | .140 | 79.550 | .163 | [-.476, .082] |
| IN X CS | .224 | .346 | 111.760 | .519 | [-.462, .911] | .323 | .445 | 113 | .469 | [-.559, 1.205] |
| IN X MD | .281 | 1.360 | 110.281 | .836 | [-2.413, 2.976] | 2.750 | 1.702 | 112.172 | .109 | [-.622, 6.121] |

**Supplementary Table 5**: *Results from Main Effects only Models*

***Table 5A****: Child-Reported Therapeutic Alliance Predicted by Child and Therapist Behavior*

|  | Relational Bond | | | | | Therapeutic Alliance | | | | |
| --- | --- | --- | --- | --- | --- | --- | --- | --- | --- | --- |
| Predictor | β | *s.e.* | *df* | *p* | 95% CI | β | *s.e.* | *df* | *p* | 95% CI |
| Individual Positive (IP) | .304 | .226 | 128.209 | .180 | [-.143, .751] | .193 | .211 | 131.455 | .362 | [-.225, .611] |
| Individual Negative (IN) | -1.065 | .437 | 132.912 | .016 | [-1.929, -.200] | -.597 | .409 | 135.479 | .146 | [-1.405, .211] |
| Group Positive (GP) | .100 | .316 | 62.436 | .753 | [-.532, .732] | -.037 | .290 | 72.905 | .898 | [-.615, .540] |
| Group Negative (GN) | .655 | .665 | 60.611 | .328 | [-.675, 1.985] | .209 | .609 | 70.675 | .732 | [-1.005, 1.423] |
| Group Management Skills (GM) | -.205 | .149 | 21.385 | .181 | [-.514, .103] | -.079 | .134 | 25.304 | .560 | [-.354, .196] |
| Clinical Skills (CS) | .056 | .432 | 22.249 | .898 | [-.839, .951] | .214 | .389 | 26.364 | .587 | [-.584, 1.012] |
| Managing Deviant Talk & Behavior (MD) | 1.366 | 1.145 | 18.895 | .248 | [-1.033, 3.764] | 1.451 | 1.028 | 22.222 | .172 | [-.680, 3.581] |

Note: Predicted variables were entered simultaneously in a single model for each outcome variable.

***Table 5B****: Therapist-Reported Therapeutic Alliance Predicted by Child and Therapist Behavior*

|  | Relational Bond | | | | | Task Collaboration | | | | | |
| --- | --- | --- | --- | --- | --- | --- | --- | --- | --- | --- | --- |
| Predictor | β | *s.e.* | *df* | *p* | 95% CI | β | *s.e.* | *df* | *p* | 95% CI |  |
| Individual Positive (IP) | 1.215 | .196 | 127.392 | <.001 | [.828, 1.602] | 1.016 | .184 | 126.104 | <.001 | [.651, 1.381] |  |
| Individual Negative (IN) | -1.535 | .360 | 126.443 | <.001 | [-2.249, -.822] | -1.541 | .339 | 125.218 | <.001 | [-2.212, -.869] |  |
| Group Positive (GP) | -.763 | .262 | 74.432 | .005 | [-1.285, -.241] | -.413 | .250 | 69.435 | .103 | [-.912, .085] |  |
| Group Negative (GN) | .367 | .513 | 62.325 | .477 | [-.659, 1.393] | .532 | .491 | 57.782 | .283 | [-.451, 1.515] |  |
| Group Management Skills (GM) | -.378 | .116 | 21.985 | .004 | [-.619, -.137] | -.493 | .113 | 20.769 | <.001 | [-.728, -.259] |  |
| Clinical Skills (CS) | .027 | .329 | 20.462 | .935 | [-.657, .712] | -.029 | .318 | 19.382 | .929 | [-.694, .637] |  |
| Managing Deviant Talk & Behavior (MD) | 1.545 | .906 | 20.445 | .103 | [-.341, 3.432] | 2.157 | .877 | 19.367 | .024 | [.323, 3.991] |  |

Note: Predicted variables were entered simultaneously in a single model for each outcome variable.

**Table 6**: *Simple Slopes for All Statistically Significant Interaction Effects*

| Figure | Individual Level Slope | Level of Moderator | | | | | | | | |
| --- | --- | --- | --- | --- | --- | --- | --- | --- | --- | --- |
|  |  | β | *s.e.* | *p* | β | *s.e.* | *p* | β | *s.e.* | *p* |
|  |  | Low Group Negative | | | Mean Group Negative | | | High Group Negative | | |
| 1 | Individual Negative | -.24 | .51 | .65 | -1.14 | .10 | <.001 | -2.05 | .27 | <.001 |
|  |  |  | | |  | | |  | | |
|  |  | Low Managing Deviant | | | Mean Managing Deviant | | | High Managing Deviant | | |
| 2 | Individual Negative | -2.67 | .77 | <.001 | -1.14 | .51 | .0262 | .38 | .69 | .58 |
|  |  |  |  |  |  |  |  |  |  |  |
|  |  | Low Group Positive | | | Mean Group Positive | | | High Group Positive | | |
| 3 | Individual Positive | 1.46 | .28 | <.001 | 1.04 | .21 | <.001 | .61 | .29 | .0385 |
|  |  |  | | |  | | |  | | |
|  |  | Low Skills | | | Mean Skills | | | High Skills | | |
| 4 | Individual Positive | .43 | .30 | .17 | 1.04 | .21 | <.001 | 1.65 | .28 | <.001 |
|  |  |  | | |  | | |  | | |
|  |  | Low Managing Deviant | | | Mean Managing Deviant | | | High Managing Deviant | | |
| 5 | Individual Negative | -2.90 | .66 | <.001 | -1.89 | .43 | <.001 | -.87 | .59 | .15 |
|  |  |  | | |  | | |  | | |
|  |  | Low Group Positive | | | Mean Group Positive | | | High Group Positive | | |
| 6 | Individual Positive | 1.44 | .26 | <.001 | .99 | .20 | <.001 | .54 | .28 | .052 |
|  |  |  | | |  | | |  | | |
|  |  | Low Skills | | | Mean Skills | | | High Skills | | |
| 7 | Individual Positive | .53 | .29 | .0688 | .99 | .20 | <.001 | 1.44 | .26 | <.001 |
